# Retraction: Fexuprazan safeguards the esophagus from hydrochloric acid-induced damage by suppressing NLRP1/Caspase-1/GSDMD pyroptotic pathway

**DOI:** 10.3389/fimmu.2026.1809767

**Published:** 2026-02-20

**Authors:** Frontiers Editorial Office

The journal retracts the 16 December 2024 article cited above.

Following publication, concerns were raised regarding the integrity of the images in the published figures. The authors failed to provide a satisfactory explanation during the investigation, which was conducted in accordance with Frontiers’ policies. As a result, the data and conclusions of the article have been deemed unreliable and the article has been retracted.

This retraction was approved by the Chief Editors of Frontiers in Immunology and the Chief Executive Editor of Frontiers. The authors were informed regarding this retraction.

